# Cleavage of Hyaluronan and CD44 Adhesion Molecule Regulate Astrocyte Morphology via Rac1 Signalling

**DOI:** 10.1371/journal.pone.0155053

**Published:** 2016-05-10

**Authors:** Anna Konopka, Andre Zeug, Anna Skupien, Beata Kaza, Franziska Mueller, Agnieszka Chwedorowicz, Evgeni Ponimaskin, Grzegorz M. Wilczynski, Joanna Dzwonek

**Affiliations:** 1 Laboratory of Molecular and Systemic Neuromorphology, The Nencki Institute of Experimental Biology, 02–093, Warsaw, ul. Pasteura 3, Poland; 2 Cellular Neurophysiology, Center of Physiology, Hannover Medical School, 30625, Hannover, Germany; 3 Laboratory of Molecular Neurobiology, Neurobiology Center, The Nencki Institute of Experimental Biology, 02–093, Warsaw, ul. Pasteura 3, Poland; Karolinska Institutet, SWEDEN

## Abstract

Communication of cells with their extracellular environment is crucial to fulfill their function in physiological and pathophysiological conditions. The literature data provide evidence that such a communication is also important in case of astrocytes. Mechanisms that contribute to the interaction between astrocytes and extracellular matrix (ECM) proteins are still poorly understood. Hyaluronan is the main component of ECM in the brain, where its major receptor protein CD44 is expressed by a subset of astrocytes. Considering the fact that functions of astrocytes are tightly coupled with changes in their morphology (e.g.: glutamate clearance in the synaptic cleft, migration, astrogliosis), we investigated the influence of hyaluronan cleavage by hyaluronidase, knockdown of CD44 by specific shRNA and CD44 overexpression on astrocyte morphology. Our results show that hyaluronidase treatment, as well as knockdown of CD44, in astrocytes result in a “stellate”-like morphology, whereas overexpression of CD44 causes an increase in cell body size and changes the shape of astrocytes into flattened cells. Moreover, as a dynamic reorganization of the actin cytoskeleton is supposed to be responsible for morphological changes of cells, and this reorganization is controlled by small GTPases of the Rho family, we hypothesized that GTPase Rac1 acts as a downstream effector for hyaluronan and CD44 in astrocytes. We used FRET-based biosensor and a dominant negative mutant of Rac1 to investigate the involvement of Rac1 activity in hyaluronidase- and CD44-dependent morphological changes of astrocytes. Both, hyaluronidase treatment and knockdown of CD44, enhances Rac1 activity while overexpression of CD44 reduces the activity state in astrocytes. Furthermore, morphological changes were blocked by specific inhibition of Rac1 activity. These findings indicate for the first time that regulation of Rac1 activity is responsible for hyaluronidase and CD44-driven morphological changes of astrocytes.

## Introduction

Astrocytes constitute the largest population of the glial cell type in the central nervous system (CNS) and play multiple supportive and regulatory roles in neuronal function [1]. They represent a heterogeneous class of cells, exhibiting different morphological appearances i.e. “fibrous” astrocytes have small cell bodies and elongated, not-branched long processes, whereas “protoplasmic” astrocytes display a bushy morphology with highly branched processes [1]. Recent studies indicate the crucial role of interactions of nerve cells and their extracellular matrix in a variety of processes such as during development, cell proliferation, synaptogenesis, synaptic transmission and plasticity, tissue injury and repair [2–8]. Astrocytes contribute to this, mainly via their responses to different stimuli from extracellular space and usually this response is accompanied by morphological changes of the astrocyte [9,10]. For instance, they acquire polarity to migrate [11] or play a role in glutamate clearance via invasion of thin astroglial processes into the synaptic cleft [12]. This astroglial synapse invasion has been recently shown to be regulated by gap junction protein connexin 30 (Cx30) [13]. Of particular importance appears to be a reaction of astrocytes responding to pathological conditions where the cells transform into a reactive state and undergo astrogliosis [14]. This process is associated with characteristic morphological changes. Cell body size of reactive astrocytes increases and their major processes become thicker, finally leading to the glial scar formation, which isolates damaged neural tissue and prevents the spread of inflammation and pathogens into the surrounding normal tissue. Though, glial scars inhibit axonal regeneration by forming chemical and mechanical barriers [14]. CD44 adhesion protein is a receptor for the main ECM component in the brain, hyaluronan [15]. Although, the expression of CD44 in astrocytes has been described [16–18], the function of the interaction of hyaluronan and its receptor in this cell type is poorly understood. In human brain, high expression of CD44 adhesion molecule has been observed in astrocytes with long, unbranched processes, whereas the cells with “protoplasmic” morphology exhibit no CD44 expression [19]. It was also shown that reactive astrocytes acquire high level of CD44 protein with changes in their morphology [20]. In other cell types, CD44 was shown to influence activity of small Rho GTPases that regulate actin cytoskeleton dynamics [21]. Furthermore, these small regulatory proteins were shown to be involved in the stellation process of astrocytes [22–26]. Here, we answer the question whether and how CD44 can regulate astrocytic shape. We examined the effect of hyaluronidase treatment on astrocyte morphology. Additionally, we have examined consequences of knockdown or overexpression of CD44 on astrocyte shape, both in conventional 2D and *in vivo*-like 3D cell cultures. Moreover, with the use of the FRET-based biosensor for Rac1 activity and dominant negative mutant of Rac1, we investigated the function of Rac1 activity in regulation of astrocyte morphology.

## Materials and Methods

### Ethics Statement

This study was carried out in accordance with the Ethical Committee on Animal Research of the Nencki Institute, based on the Polish Act on Animal Welfare and other national laws that are in full agreement with EU directive on animal experimentation. The protocols were approved by the Committee on the Ethics of Animal Experiments of the Nencki Institute. Pregnant females of Wistar rat were housed 1 per cage in a controlled room temperature (22°C ± 1°C) under a 12-hour dark/light cycle (lights off at 10 a.m.) with free access to water and food in the Animal House of The Nencki Institute. 0-5-days-old rat pups were sacrificed by decapitation and the brains were removed.

### DNA constructs

The following mammalian expression plasmids were used in the study and have been described previously: pSuper vector [27], β-actin-GFP [28], β-actin-RFP [29] (vectors encoding GFP or RFP proteins under the control of β-actin promoter), CD44-GFP/RFP, CD44shRNA, CD44Rescue [18], pcDNA3-EGFP-Rac1-T17N (Rac1-DN, dominant negative Rac1) and FRET-based biosensor Raichu-Rac1/1011x [30]. Rac1-DN was a gift from Gary Bokoch (Addgene plasmid # 12982, [31]). The FRET based biosensors pRaichu1011X(Rac1), Raichu-RhoA and Raichu-Cdc42 [30] were kindly provided by Prof M. Matsuda (Department of Pathology and Biology of Diseases, Kyoto University, Japan). To obtain the reference spectra the Venus/pCS2 and pECFP constructs were used.

### Cell cultures and transfection

Primary rat astrocytes cultures were prepared from P0-P5 Wistar rat brains as described previously with slight modifications [32]. Briefly, cells were isolated from cerebral cortices by trypsinization (0.025% trypsin at 37°C for 20 min) and mechanically dissociated to a single cell suspension. The cells were suspended in medium containing 10% FBS in DMEM (high glucose) with 1% penicillin and streptomycin and transferred to 75 cm^2^ culture flasks. Fresh medium was provided after 4 days. As the cells became confluent, normally within 7–9 days, flasks were shaken at 200 rpm for 24h at 37°C to remove microglial and oligodendrocytic cells. Afterwards, medium was exchanged. The shaking step was repeated after 4–5 days. Astrocytes were transfected by electroporation with the use of Basic Nucleofector Kit for Mammalian Glia Cells according to the manufactural protocol (Lonza). After transfection cells were plated on 12mm glass cover slips in 24-well plates pre-coated with poly-L-lysine (Sigma) and cultured at 37°C in a 95% air/5% CO^2^ incubator. In case of 3D cell cultures, the astrocytes were seeded on 24-well plates with bioactive 3D inserts (3Dtro AB) [33].

### Immunofluorescence

The procedures were performed on cultured cells as described previously [34]. The polyclonal sheep anti-CD44 primary antibody (1:500, R&D, catalog number AF6577) and polyclonal donkey anti-sheep Alexa Fluor 555 (1:500, Life technologies, catalog number A21436) were used. In case of hyaluronan binding protein (HABP) staining, cells were fixed, washed with 1xPBS and blocked with 5% normal donkey serum. Subsequently cells were blocked with Avidin/Biotin Blocking Kit (Vector) according to the manufactural protocol. Next, cells were incubated overnight with HABP coupled with biotin (1:200, Sigma) at 4°C. After three washes with 1xPBS, cells were incubated with Avidin conjugated with Alexa Fluor 555 (Invitrogen), washed, and mounted. Nuclei were stained with 4,6-diamidino-2-phenylindole (DAPI) (VECTASHIELD Mounting Medium with DAPI, VECTOR).

### Förster Resonance Energy Transfer (FRET) investigations

The FRET measurements were performed as described previously by Duhr et. al [35]. FRET images were acquired using a Carl Zeiss LSM780 confocal microscope. The reference CFP emission spectrum was acquired from 436 to 694 nm with a 8.75 nm precision using 445 nm excitation in a CFP-transfected cell. The YFP spectrum was acquired from a YFP-transfected cell image (458 nm excitation and same spectral range). The images of cells expressing Raichu-Rac1/1011x, Raichu-Cdc42/1054x, Raichu-RhoA probes were acquired (445 nm excitation, same spectral range). The pixel dwell time was 1–3 μs to avoid unwanted noise. Spectral separation was performed using Matlab weighted unmixing algorithm with background correction and the reference spectra of CFP and YFP alone. Biosensor readout was calculated at pixel basis from the ratio of YFP to CFP intensity obtained from spectral unmixing. The ratio of the YFP to CFP signals reflects the Rac1 activation, where high ratio refers to active and low to inactive state of the protein.

For the quantitative analysis of FRET data, novel evaluation algorithm [36] were used which were successfully adapted to quantify FRET signal at the single-cell level [37,38].

### Hyaluronidase treatment

Astrocytes cultured *in vitro* were starved overnight in medium without serum and then incubated for 24h with hyaluronidase type IV-S (100mU/ml, Sigma), heat-inactivated hyaluronidase or deionized sterile water.

### Image acquisition and morphometric analysis

Fluorescently labeled cells were examined using Zeiss LSM 780 confocal microscope. The analysis of the average immunofluorescence intensity of CD44 and the used morphometric factors for cell shape were performed using ImageJ software [39]. The fluorescence intensity of Alexa Fluor 555 in transfected cells was normalized to the intensity of non-transfected adjacent cells. Three factors describing cell shape were used: circularity, solidity and area. The circularity is described by 4π×[Area]/[Perimeter]^2^ with a value of 1.0 indicating a perfect circle. The solidity is described by [Area]/[Convex area], where convex area is a section defined by a band wrapped tightly around the points. The values for solidity and circularity increase when the cell shape deviates from the stellate-like morphology. Additionally, the branching factor was calculated as a number of astrocytic prtrusions.

### Statistical analyses

Data show mean values and standard error of the mean and were analyzed using one-way analysis of variance (ANOVA) followed by Dunnett’s C [40] or Sidak [41] post hoc tests, depending on whether homogeneity of variance was assumed, and t-Student test. From 20 to 50 cells per group were measured. The cells were obtained from three independent batches of astrocytes. The statistical analyses were performed using IBM SPSS software.

## Results

### Hyaluronan (HA) digestion induces morphological changes of astrocytes

**Fig 1:**
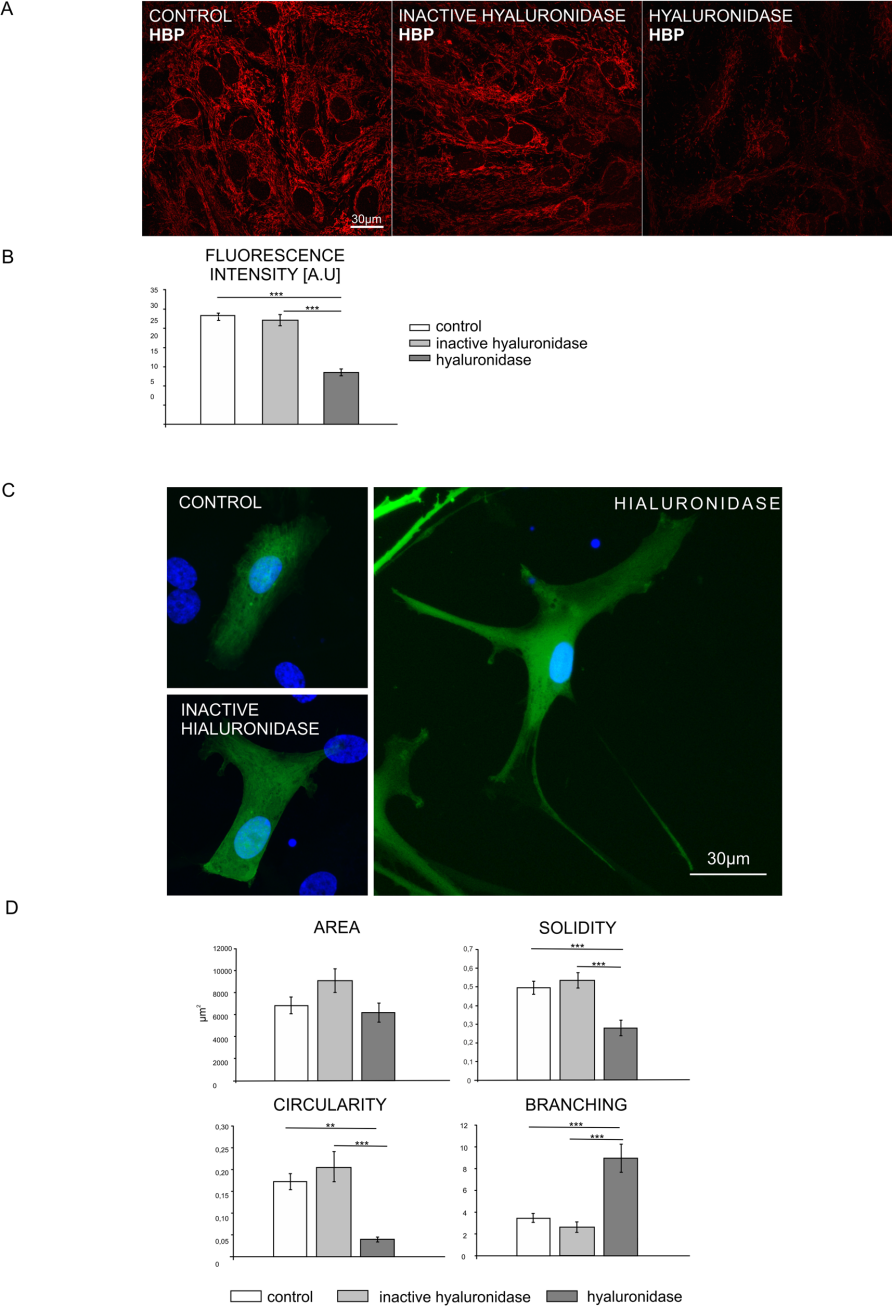
Astrocytes treated with hyaluronidase acquire the stellate-like morphology. A. Hyaluronan digestion by hyaluronidase was evaluated by staining with hyaluronan binding protein (HABP) (red). Scale: 30 μm. B. Measurement of fluorescence intensity. One way ANOVA test was performed, F(2.57) = 53.169; p<0.001, Dunnett’s C post hoc. C. Representative images of astrocytes transfected with β-actin GFP and either untreated (control) or treated with hyaluronidase or heat inactivated hyaluronidase for 48h. Cell nuclei were visualized with DAPI staining. Scale: 30 μm.D. Morphometric analysis of shape-describing parameters of cells treated as described in C. One way ANOVA test was performed, area: F(2.57) = 2.658; p>0.05, solidity: F(2.57) = 16.814; p<0.001, circularity: F(2.57) = 13.799; p<0.001, branching: F(2.57) = 16.774; p<0,001 Dunnett’s C post hoc test.

To investigate whether HA regulates astrocyte morphology we investigated the effect of hyaluronidase treatment on astrocytes cultured *in vitro*. Astrocytes were transfected with a β-actin-GFP plasmid (encoding green fluorescent protein under the control of a β-actin promoter) for visualization of single cell morphology. Four days after transfection, cells were starved overnight in medium without serum and were incubated or not with active or heat-inactivated hyaluronidase for 48h. In order to evaluate the efficiency of HA digestion by hyaluronidase, astrocytes were stained with hyaluronan binding protein (HABP) (Fig 1A). Hyaluronidase treatment significantly decreased the level of the HABP staining comparing to controls, indicating that the hyaluronan was effectively degraded by the enzyme (Fig 1B). However, it should be noticed that our experimental approach does not permit to completely rule out the possibility that bovine testes hyaluronidase can cleave hyaluronan into oligosaccharides that can not be recognized by HABP [42], but may influence cellular signaling [43]. Astrocytes treated with hyaluronidase acquired the stellate-like morphology with numerous thin protrusions extending from the cell body (Fig 1C). To quantitatively describe the observed changes we performed morphometric analysis. Three different shape-describing parameters, namely area, solidity and circularity and branching were measured with the use of ImageJ software. Solidity and circularity parameters were significantly lower and branching was increased in hyaluronidase treated astrocytes in comparison to controls, confirming “stellation” of the astrocytes upon hyaluronidase treatment (Fig 1D).

### CD44 regulates morphology of astrocytes

**Fig 2:**
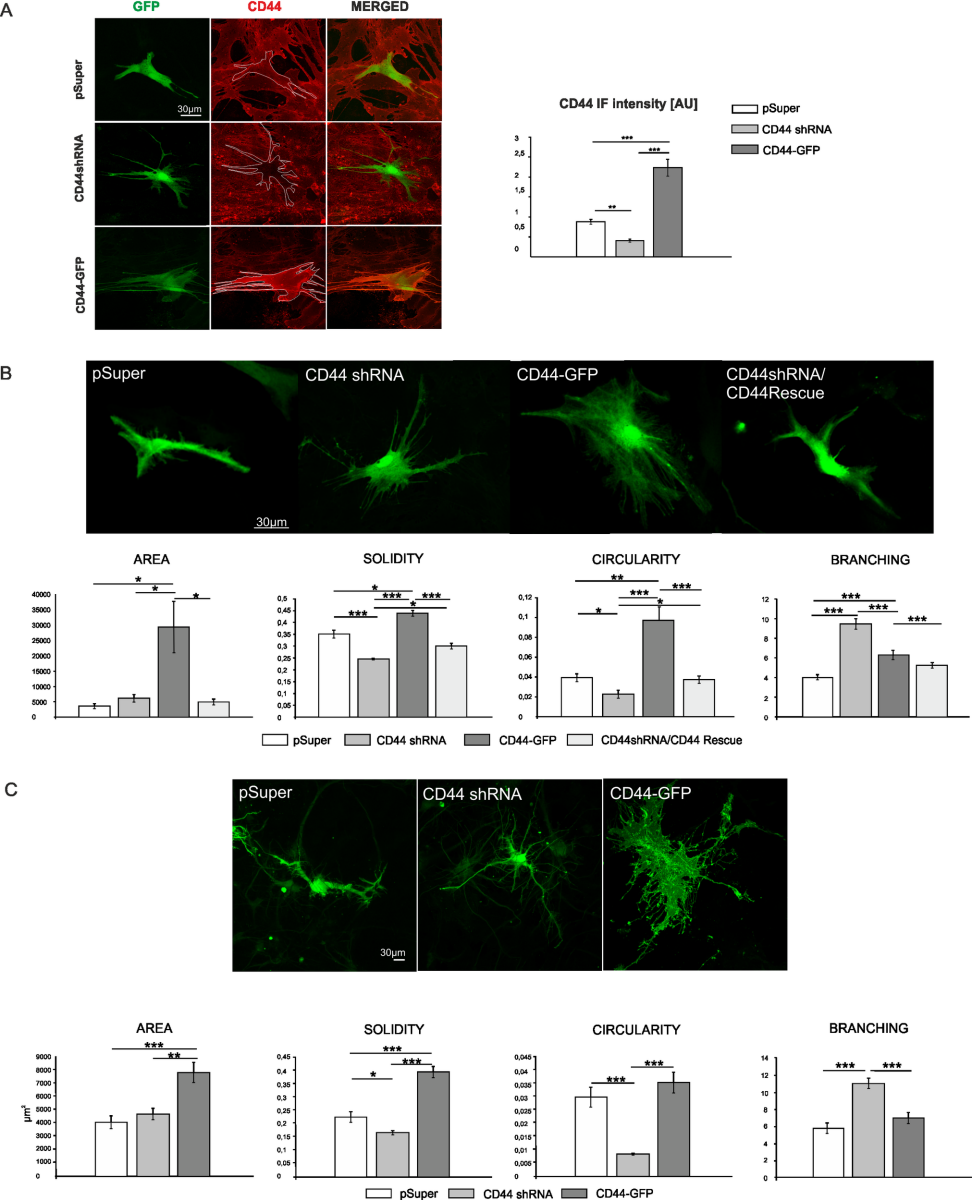
CD44 regulates astrocyte morphology. A: Validation of CD44shRNA and CD44-GFP constructs. Astrocytes were transfected with pSuper, CD44shRNA or CD44-GFP constructs (together with β-actin-GFP plasmid) and then immunostained with anti-CD44 antibody (red). The level of CD44 expression was evaluated by measuring CD44 immunofluorescence (IF) signal intensity with the use of ImageJ program. One way ANOVA, F(2.71) = 71.187, p<0.001, Dunnett C post hoc tests. Scale: 30 μm. B: Morphological analysis of shape-describing parameters of astrocytes in 2D cultures co-transfected with pSuper, CD44shRNA or CD44shRNA/CD44Rescue constructs together with β-actin-GFP plasmid. One way ANOVA test was performed, area: F(3.150) = 8.169; p<0.001, solidity: F(3.153) = 21.454; p<0.001, circularity: F(3.153) = 18.873; p<0.001, Dunnett’s C post hoc tests, branching: F(3.151) = 33,478; p<0.001, Sidak post hoc test. Scale: 30 μm. C: The morphological analysis of shape-describing parameters of astrocytes in 3D cultures transfected with pSuper or CD44shRNA constructs (together with β-actin-GFP plasmid) or CD44-GFP. One way ANOVA test was performed, area: F(2.92) = 12.311; p<0.001, Sidak post hoc test; solidity: F(2.95) = 42.208; p<0.001, Dunnett’s C post hoc test, circularity: F(2.94) = 20.609; p<0.001, Dunnett’s C post hoc test, branching: F(2.95) = 17.703; p<0.001, Sidak post hoc test. Scale: 30 μm.

To determine the function of CD44 in astrocytes, we examined the effects of CD44 knockdown or CD44 overexpression on astrocyte morphology. The high efficiency of shRNA constructs used to deplete CD44 expression was recently shown in HEK-293 cells and in hippocampal neurons [18]. We tested the efficacy of this shRNA in cultured astrocytes. Cells were transfected with an empty pSuper vector (control) or CD44 shRNA plasmid. The β-actin-GFP plasmid was added to the transfection mixture for the identification of transfected cells. To increase the level of CD44 in astrocytes we used CD44-GFP encoding plasmid. Four days after transfection, CD44 was detected by immunofluorescence, and the intensity of immunostaining was measured with ImageJ software. As shown in Fig 2A CD44 shRNA prominently and significantly decreased, whereas CD44-GFP increased the level of CD44 protein. We observed that a low level of CD44 expression promotes stellate-like morphology of cells without significant changes in the area of the cells, an effect similar to that observed upon hyaluronidase treatment (Fig 2B). In contrast, the upregulation of CD44 expression leads to an increased cell area and flattened morphology. To confirm the specificity of the observed CD44 knockdown phenotype in astrocytes, a rescue experiment was performed. We used CD44Rescue construct in which silent mutations were introduced into the cDNA coding region for rat CD44, which was transcribed into mRNA that could not be recognized by shRNA. The co-expression of CD44Rescue with CD44shRNA in astrocytes resulted in the partial reversal of the knockdown-induced phenotype (Fig 2B). These data indicate that CD44 shRNA-induced changes in astrocytes morphology resulted from the specific knockdown of CD44 rather than from off-target effects. The effects of altered CD44 protein level on astrocytes were also investigated in 3D cell cultures. In this type of culture, the cells grow on randomly oriented polyurethane fiber scaffolds, coated with a special composition of bioactive molecules, allowing astrocytes to grow more freely according to their own metabolic programs [33]. In 3D cultures, CD44 knockdown, shows the same effects on astrocyte morphology as observed in 2D cultures (Fig 2C). The values of area, solidity, circularity and branching parameters undergo changes similar to 2D cultures, except the circularity values for cells transfected with CD44-GFP vector. These findings show that the role of CD44 in the regulation on cell morphology is specific and not restricted to the conditions in 2D standard cell culture.

### Morphological changes of astrocytes induced by hyaluronidase treatment and CD44 depletion depend on Rac1 activity

**Fig 3:**
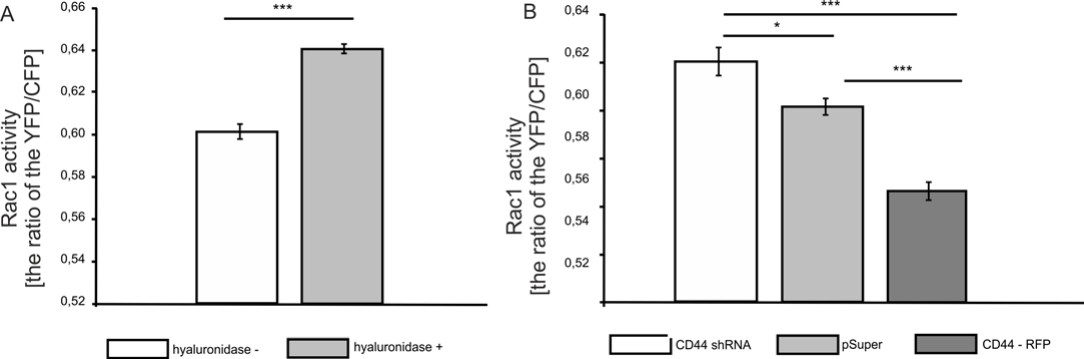
Hyaluronidase treatment and CD44-knockdown leads to enhanced Rac1 activity. A: Cells were transfected with FRET based biosensor pRaichu-Rac1/1011X and then treated or not with hyaluronidase for 24h. YFP-CFP ratio was calculated as a readout of Rac1 activity. T-Student test, t(589) = 3.212; p<0,001. B: Cells were co-transfected with pRaichu-Rac1/1011X and pSuper/CD44shRNA/CD44-RFP constructs. YFP-CFP ratio was calculated as a readout of Rac1 activity. One way ANOVA, F(2.527) = 39.998; p<0.001, Sidak post hoc test.

In order to determine whether the small GTPase Rac1 could be responsible for the morphological changes of astrocytes after hyaluronidase treatment, FRET technique was applied. A FRET-based biosensor for Rac1 (Raichu-Rac1/1011x) which allows measurement of Rac1 activity was used [30]. Astrocytes were transfected with Raichu-Rac1/1011x biosensor and analyzed after hyaluronidase or control incubation. Rac1 activation was observed after hyaluronidase treatment compared to non-treated control cells (Fig 3A). Furthermore, the influence of knockdown of CD44 and CD44 overexpression on Rac1 activity were investigated. In this case, astrocytes were co-transfected with Raichu-Rac1/1011x biosensor together with CD44shRNA or CD44-RFP. We observed an elevated level of Rac1 activity in CD44shRNA-transfected cells, whereas CD44-RFP-transfection resulted in a reduced Rac1 activity (Fig 3B).

The effects of each individual Rho GTPase on astrocyte morphology depend also on the relative level of expression and activity of other GTPases in the cell. To address the question whether RhoA and Cdc42 activity are also changed by hyaluronidase treatment or altered CD44 expression, we performed similar experiments with the use of Raichu-RhoA and Raichu-Cdc42 biosensors. The results indicate that hyaluronan digestion by hyaluronidase induces activity of RhoA whereas decreases activity of Cdc42 (S1A and S1C Fig). What is interesting, the activity of Cdc42 was decreased in CD44-knocked down as well as CD44-overexpressing cells (S1B Fig). In turn, the activity of RhoA was decreased in CD44-depleted astrocytes, but was not changed by CD44 overexpression (S1D Fig).

**Fig 4:**
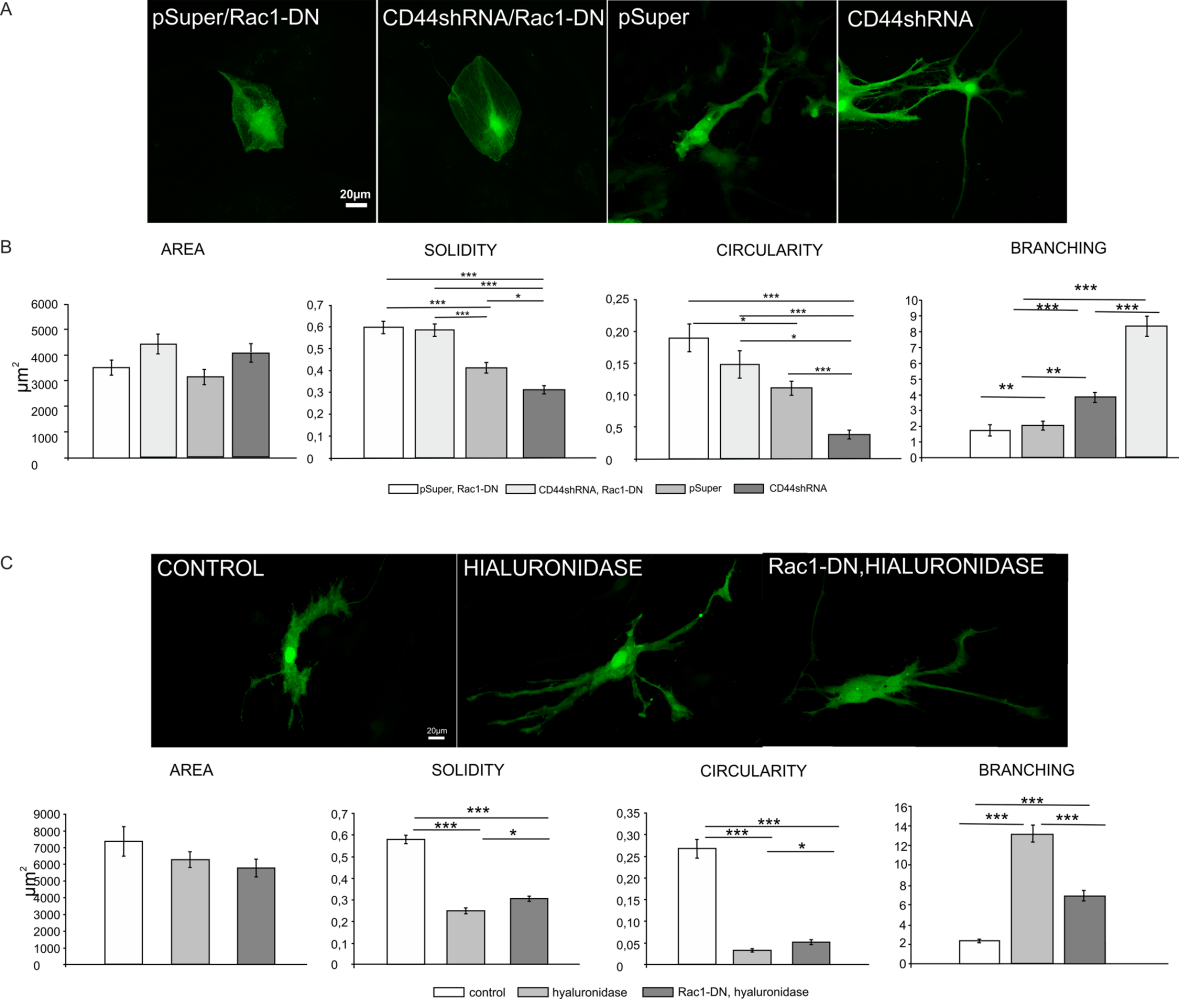
Deactivation of Rac1 activity rescues CD44 knockdown and hyaluronidase-induced morphological changes of astrocytes. A: Representative images of astrocytes transfected with CD44shRNA/pSuper or co-transfected with pcDNA3-EGFP-Rac1-T17N (Rac1-DN) constructs. The β-actin-RFP construct was used for cell visualization. Scale: 20 μm. B: Morphometric analysis of shape-describing parameters of cells treated as in A. One way ANOVA, area: F(3.112) = 2.456, p>0,05, solidity: F(3.114) = 30.173, p<0.001 Sidak post hoc test, circularity: F(3.114) = 13.834, p<0.001, branching: F(3.114) = 51, 825, p<0,001. Dunnett’s C post hoc test. C: Representative images of astrocytes transfected with pcDNA3-EGFP-Rac1-T17N (Rac1-DN) and β-actin-RFP constructs and treated with hyaluronidase. Scale: 20 μm. D: Morphometric analysis of shape-describing parameters of cells treated as in C. One way ANOVA, area: F(2.147) = 1.520, p>0.05, solidity: F(2.147) = 106.292, p<0.001, circularity: F(2.147) = 96.843, p<0.001, branching: F(2.147) = 135.932; p<0,001, Dunnett’s C post hoc tests.

To check whether activation of Rac1 is responsible for hyaluronidase and CD44shRNA-induced astrocyte stellation, we blocked Rac1 activity by introducing the pcDNA3-EGFP-Rac1-T17N dominant-negative mutant (see M&M) into the cells. Astrocytes were co-transfected with either pSuper, or CD44shRNA together with Rac1-DN plasmid (Fig 4A and 4B) or co-transfected with β-actin-GFP and Rac1-DN and then treated with hyaluronidase for 24h (Fig 4C and 4D). Morphometric analysis revealed that CD44shRNA-induced stellation was effectively inhibited in cells transfected with Rac1-DN (Fig 4B). The deactivation of Rac1 also blocked hyaluronidase-induced stellation, but to a lower extent (Fig 4D). This shows, that Rac1 is required to develop a stellate-like morphology of astrocytes. Taken together, these experiments demonstrate the roles of 1) hyaluronan cleavage, 2) the expression level of its receptor CD44, and 3) the activity of their downstream effector Rac1 in the regulation of the astrocyte morphology.

## Discussion

In this study, we investigated the role of hyaluronan, CD44 adhesion molecule and associated cellular signaling events regulating astrocyte morphology. We demonstrated, for the first time, that stellation of astrocytes in 2D and 3D cultures *in vitro*, is caused by CD44 inhibition and hyaluronan digestion by hyaluronidase. Moreover we showed that the observed hyaluronan/CD44-dependent morphological changes of astrocytes depend on Rac1 activity.

ECM proteins play an important role in physiology and pathology of the nervous system influencing all cell types including astrocytes [44]. The synthesis of hyaluronic acid and CD44 are elevated in a variety of brain pathologies [45–51]. Hyaluronan accumulates in injured spinal cord, as gliosis proceeds, and maintains the astrocytes in a state of quiescence. Then the hyaluronidase, that is induced soon after spinal cord injury, leads to HA degradation and increased astrocyte proliferation [52]. This finding demonstrates that hyaluronan plays an inhibitory role in astrocyte proliferation. On the other hand, our observation that hyaluronidase treatment induces astrocyte stellation suggests that HA can inhibit formation of protrusions of astrocytes. Our experiments with shRNA, which specifically deplete CD44 expression, indicate that hyaluronan exerts its inhibitory role through binding to CD44. Accumulation of perisynaptic ECM molecules is believed to restrain invasion of thin astrocytic processes into the synaptic cleft, and hereby regulate synaptic function [44,53].

The process of astrocyte stellation depends on activation of Rac1 and cytoskeleton rearrangements [22]. The clarified function of CD44 in regulation of the actin cytoskeleton [54] is consistent with our findings describing its role in the control of astrocyte morphology. We show that hyaluronidase treatment induces Rac1 activity in astrocytes. Moreover, we show that astrocyte stellation induced by hyaluronidase treatment depends on Rac1 activity since the effect is abolished upon expression of a dominant negative Rac1 mutant. Rac1 activity has not been investigated upon hyaluronidase treatment so far. In contrast, previous reports show that hyaluronan or its interaction with CD44 induce Rac1 activity in various cell types including astrocytes, however in a different experimental paradigm. This HA-induced Rac1 activity leads to enhanced astrocyte migration and promotes tumor progression [11,55].

The observed effects of hyaluronidase can be induced either by the breaking of the link between cells and ECM or by the degradation of HA into short fragments (LMW low molecular weight) that can interact with membranous receptors and trigger different effects than long HA polymers. Consistently, we observed similar effects when we silenced CD44 and the opposite effects were induced by CD44 overexpression. There are a number of possible mechanisms that might explain Rac1 activation in the absence of CD44. The relative level of expression and activity of different GTPases in the cell is responsible for regulation of astrocyte morphology by individual RhoGTPase. As we show on S1 Fig, the increase in the Rac1 activity in CD44-depleted cells, is accompanied by a simultaneous decrease in the activity of RhoA. It is known that the activation of RhoA can cause inhibition of Rac1 in many cell types [56]. Thus, the decrease of RhoA activity could contribute to decreased inhibition of Rac1 activity. A similar pattern of expression of small RhoGTPases we also observed in the dendritic spines of neurons with a diminished expression of CD44 [57].

Another possibility is, that CD44 protein can influence RhoGTPase’s activity through interaction with guanine nucleotide exchange factors (GEFs) that can activate Rac1 e.g. TIAM1 (T-Cell Lymphoma Invasion And Metastasis 1) [58] or Vav2 (Vav2 Guanine Nucleotide Exchange Factor) [59], as well as with their inhibitors (GAPs, GTPase-activating proteins) e.g. IQGAP1 (IQ Motif Containing GTPase Activating Protein 1) [60]. The removal of the CD44 from the cell may result in reduced amount of Rac1 specific GAPs, which in turn leads to the predominance of activation of GEFs by other mechanisms in the cell. This allows to regulate Rac1 activity in different manners depending on the cellular process that is controlled by HA/CD44 interaction and is in line with the notion about a dual role of CD44 in tumor cells [61].

Our results also indicate that hyaluronan digestion by hyaluronidase decreases activity of Cdc42. What is interesting, but difficult to interpret the activity of Cdc42 was decreased in CD44-knocked down as well as CD44-overexpressing cells. The complexity of this whole system is underscored by our unpublished data showing that Cdc42 is activated upon CD44 silencing in dendritic spines of neurons [57]. The opposite effects of CD44 depletion can be cell-specific and can be, among others, related to the significantly different levels of CD44 expression in astrocytes and neurons.

In turn, the activity of RhoA was decreased in CD44-depleted astrocytes, but was not changed by CD44 overexpression. However, hyaluronidase treatment caused the induction of RhoA activity. Taken together, these results indicate that the effects on RhoGTPase’s activity in astrocytes evoked by HA digestion or by changes in CD44 expression overlap only partially. This is also in agreement with the observation that morphological effects induced by hyaluronidase are less reversible by Rac1DN mutant than those caused by altered CD44 expression (Fig 4).

The morphology of astrocytes cultured *in vitro* differs dependently on the type of the culture. To ensure that the results that we obtained using conventional 2D cultures is not only specific for this type of culture, we also used a three-dimensional cell culture system. Astrocytes grown on such scaffold exhibit morphological features of *in vivo* astrocytes [33]. We measured the changes in morphology of astrocytes with three different parameters that describe the cell shape. The results obtained for 2D and 3D-cultured astrocytes were similar, suggesting that CD44 can regulate astrocyte shape not only *in vitro*.

In summary, our findings show that receptor CD44 enables astrocytes to respond to extracellular signals and decipher them into intracellular signaling pathways, to maintain and modulate their morphology. This mechanism can underlie the morphological changes associated with many physiological and pathological processes in the CNS.

## Supporting Information

S1 Fig The effects of Cdc42 and RhoA on astrocytes morphology. A: Cells were transfected with FRET based biosensor pRaichu-Cdc42/1054X and then treated or not with hyaluronidase for 24h. YFP-CFP ratio was calculated as a readout of Cdc42 activity. Mann Whitney test, p<0,001. B: Cells were co-transfected with pRaichu-Cdc42/1054X and pSuper/CD44shRNA/CD44-RFP constructs. YFP-CFP ratio was calculated as a readout of Cdc42 activity. One way ANOVA, F(2.297) = 4.195; p<0.01, Dunnett’s C post hoc test. C: Cells were transfected with FRET based biosensor pRaichu-RhoA/1237X and then treated or not with hyaluronidase for 24h. YFP-CFP ratio was calculated as a readout of RhoA activity. Mann Whitney test, p<0,01. D: Cells were co-transfected with pRaichu-RhoA/1237X and pSuper/CD44shRNA/CD44-RFP constructs. YFP-CFP ratio was calculated as a readout of RhoA activity. One way ANOVA, F(2.296) = 43.370; p<0.001, Dunnett’s C post hoc test.
(TIF)
