# Supplementary figures and images for: Cleavage of Hyaluronan and CD44 Adhesion Molecule Regulate Astrocyte Morphology via Rac1 Signalling
Source: PLoS One. 2016 May 10;11(5):e0155053. doi: 10.1371/journal.pone.0155053 (PMC4862642; doi:10.1371/journal.pone.0155053)

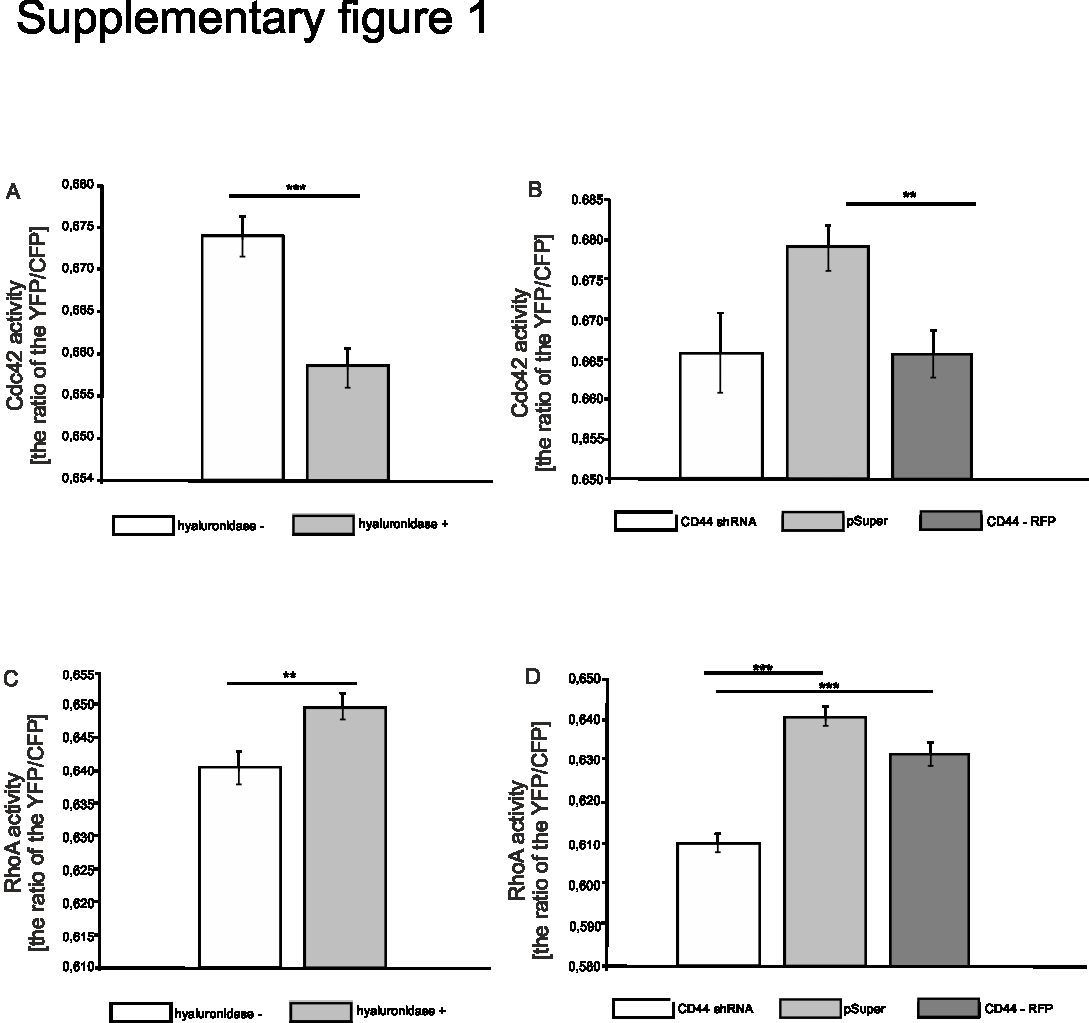

Supplement: S1 Fig — A: Cells were transfected with FRET based biosensor pRaichu-Cdc42/1054X and then treated or not with hyaluronidase for 24h. YFP-CFP ratio was calculated as a readout of Cdc42 activity. Mann Whitney test, p<0,001. B: Cells were co-transfected with pRaichu-Cdc42/1054X and pSuper/CD44shRNA/CD44-RFP constructs. YFP-CFP ratio was calculated as a readout of Cdc42 activity. One way ANOVA, F(2.297) = 4.195; p<0.01, Dunnett’s C post hoc test. C: Cells were transfected with FRET based biosensor pRaichu-RhoA/1237X and then treated or not with hyaluronidase for 24h. YFP-CFP ratio was calculated as a readout of RhoA activity. Mann Whitney test, p<0,01. D: Cells were co-transfected with pRaichu-RhoA/1237X and pSuper/CD44shRNA/CD44-RFP constructs. YFP-CFP ratio was calculated as a readout of RhoA activity. One way ANOVA, F(2.296) = 43.370; p<0.001, Dunnett’s C post hoc test. (TIF) [file pone.0155053.s001.tif]
